# Kaposi’s sarcoma-associated herpesvirus ORF61 protein sequesters APOBEC3B in filamentous aggregates

**DOI:** 10.1128/jvi.00789-25

**Published:** 2025-06-05

**Authors:** Laura-Marie Luoto, Enrico Caragliano, Carola Schneider, Rudolph Reimer, Wolfram Brune

**Affiliations:** 1 Leibniz Institute of Virology (LIV) 28367, Hamburg, Germany; 2 Centre for Structural Systems Biology (CSSB), Hamburg, Germany; 3 Institute of Virology, Hannover Medical School 686461, Hannover, Germany; The University of Arizona, Tucson, Arizona, USA

**Keywords:** KSHV, human herpesvirus 8, MHV-68, ribonucleotide reductase, aggregate, fibril, FRAP, CLEM

## Abstract

Herpesviruses are large DNA viruses that encode homologs of cellular enzymes. The viral ribonucleotide reductase, which consists of large R1 and small R2 subunits, is required for deoxyribonucleotide synthesis. However, herpesviruses have repurposed the R1 subunit for additional non-canonical functions in virus-host interaction and immune evasion. Here, we investigated the R1 proteins of Kaposi’s sarcoma-associated herpesvirus (KSHV) and murine gammaherpesvirus 68 (MHV-68), two γ-herpesviruses of the genus *Rhadinovirus*. We show that the ORF61-encoded viral R1 proteins form elongated cytoplasmic condensates in infected cells, which structurally differ from the previously described R1 condensates of other herpesviruses. Fluorescently labeled ORF61 condensates exhibited the properties of solid aggregates, as determined by fluorescence recovery after photobleaching (FRAP). Correlative light and electron microscopy (CLEM) showed that ORF61 aggregates consist of filamentous bundles. The KSHV ORF61 protein interacted with the cellular cytosine deaminase APOBEC3B in infected cells and translocated it from the nucleus, the site of viral DNA replication, to the cytoplasmic aggregates. Aggregate formation and relocalization of APOBEC3B depended on a conserved Induced Protein Aggregation Motif (IPAM) in the C-terminal part of ORF61. A KSHV ORF61 IPAM mutant was vulnerable to APOBEC3B-mediated deamination and replicated to reduced titers. In contrast, MHV-68 ORF61 did not relocalize human or murine APOBEC3 proteins, suggesting that it engages different target proteins. The results show that rhadinovirus ORF61 proteins form elongated filamentous aggregates in infected cells to sequester and inactivate target proteins, such as APOBEC3B.

**IMPORTANCE:**

Herpesviruses are large DNA viruses that encode enzymes similar to those in host cells. The R1 subunit of their ribonucleotide reductase is important for DNA synthesis and plays additional roles in immune evasion and virus-host interactions. This study focused on the R1 protein ORF61 of two γ-herpesviruses of the genus *Rhadinovirus*: KSHV and MHV-68. Unlike their homologs in other herpesviruses, KSHV and MHV-68 R1 proteins form cytoplasmic aggregates consisting of filamentous bundles in infected cells. KSHV ORF61 depletes the mutagenic cellular enzyme APOBEC3B from the nucleus, the site of viral DNA replication, and sequesters it in cytoplasmic aggregates, thereby protecting the viral genome from APOBEC3B-mediated mutations. This process relies on a specific conserved motif in ORF61. However, the MHV-68 ORF61 protein does not redistribute APOBEC3 proteins, suggesting that it binds different targets. These findings reveal how rhadinoviruses use filamentous ORF61 aggregates to manipulate host antiviral defenses.

## INTRODUCTION

Herpesviruses have large dsDNA genomes that contain up to 200 canonical open reading frames (ORFs). They encode a great number of proteins and polypeptides, several of which are homologous to cellular proteins. Two such proteins are the large and small subunits of ribonucleotide reductase (RNR), an enzyme catalyzing the conversion of ribonucleotides to deoxyribonucleotides (dNTPs). The viral RNR facilitates viral replication in cells with low dNTP pools (1, 2). Whereas herpesviruses of the α- and γ-subfamilies encode a functional heterotetrameric RNR consisting of two large catalytic R1 subunits and two small regulatory R2 subunits, the β-herpesviruses encode only a catalytically inactive R1 and lack an R2 protein (3, 4). Interestingly, herpesviruses have repurposed the R1 protein to carry out additional functions beyond dNTP synthesis. The R1 protein M45 of murine cytomegalovirus (MCMV) interacts with receptor-interacting protein kinase 1 (RIPK1) and nuclear factor (NF)-κB essential modulator (NEMO) to prevent necroptosis and NF-κB-mediated inflammatory signaling (5–7). M45 achieves this by sequestering RIPK1 and NEMO in bulky cytoplasmic aggregates that are subsequently targeted for degradation by selective autophagy (6, 8). This process is largely conserved in herpes simplex virus type 1 (HSV-1), an α-herpesvirus, that uses its R1 protein ICP6 to induce RIPK1 aggregation and degradation in a similar manner (8). M45 and ICP6 aggregation and sequestration of target proteins require a C-terminal Induced Protein Aggregation Motif (IPAM) that is conserved in more than 70 viral R1 protein homologs not only across the three herpesvirus subfamilies but also in baculoviruses and giant viruses (8, 9). Although the IPAM is also present in γ-herpesviruses, its functional relevance in γ-herpesvirus infection has not been investigated. Furthermore, it is unknown whether γ-herpesviruses, similar to MCMV and HSV-1, employ R1-mediated aggregation and degradation of cellular target proteins to counteract host defenses.

The γ-subfamily of herpesviruses has two human pathogenic members that are both oncogenic: EBV, a γ1-herpesvirus of the genus *Lymphocryptovirus*, and Kaposi’s sarcoma-associated herpesvirus (KSHV, also known as human herpesvirus 8), a γ2-herpesvirus of the genus *Rhadinovirus* (10). EBV is associated with B-cell lymphomas and nasopharyngeal and gastric carcinomas, whereas KSHV has a causative role in Kaposi’s sarcoma, an AIDS-defining malignancy of endothelial origin, as well as in primary effusion lymphoma and multicentric Castleman’s disease, two lymphoproliferative disorders (11). Since EBV and KSHV do not infect laboratory animals, the murine gammaherpesvirus 68 (MHV-68), a rhadinovirus isolated from rodents, is frequently used as a model to study KSHV pathogenesis (12, 13).

Among the γ-herpesvirus R1 proteins, EBV BORF2 has been most extensively characterized. BORF2 interacts with the cellular enzyme APOBEC3B (A3B), inhibits its catalytic activity, and relocalizes it from the nucleus to the cytoplasm upon reactivation from latency and induction of the lytic cycle, thereby protecting the replicating viral genome from detrimental mutations (14). Although the structure of purified EBV BORF2 bound to A3B has been resolved using cryo-electron microscopy (cryo-EM) (15), the physical properties of the cytoplasmic condensates, in which both proteins accumulate in infected cells, have not been further characterized. A similar mechanism of R1-dependent A3B relocalization has been demonstrated in HSV-1-infected cells (16, 17).

Human A3B belongs to the APOBEC3 (A3) protein family of seven single-stranded DNA cytosine deaminases (A3A to D and F to H) that originate from structural reorganization events in the ancestral A3 gene locus in placental mammals (18–20). A3 proteins are cellular innate immune effectors that convert cytosine bases to uracil, thereby exhibiting a mutagenic effect on viral genomes (21). Besides EBV and HSV-1, A3 family proteins are restriction factors for several different viruses, such as HIV-1 and HIV-2, hepatitis B virus, papilloma- and polyomaviruses, all of which have developed strategies to counteract A3-mediated restriction (22–24). Interestingly, only one A3 gene is present in the mouse genome, and the mA3 protein is a known restriction factor for murine retroviruses (22, 25).

Whereas the structure and function of the EBV R1 protein, BORF2, have been studied in great detail (14, 15), very little is known about non-canonical functions of rhadinovirus R1 proteins. Therefore, we decided to investigate the R1 proteins of KSHV and MHV-68 (encoded by the viral ORF 61) during viral infection, their ability to form condensates, and to sequester target proteins. We show that the ORF61 proteins of KSHV and MHV-68 form large elongated aggregates in infected cells that are not degraded by autophagy. These aggregates are composed of filamentous bundles, as determined by EM, and their formation requires the C-terminal IPAM motif. KSHV ORF61 relocalizes and sequesters A3B in these cytoplasmic filamentous aggregates in an IPAM-dependent manner to prevent deamination of the viral genome, whereas MHV-68 ORF61 does not relocalize murine A3 proteins, suggesting that it may sequester other as-yet unknown cellular target proteins.

## RESULTS

### Rhadinovirus R1 proteins accumulate in elongated cytoplasmic condensates

**Fig 1:**
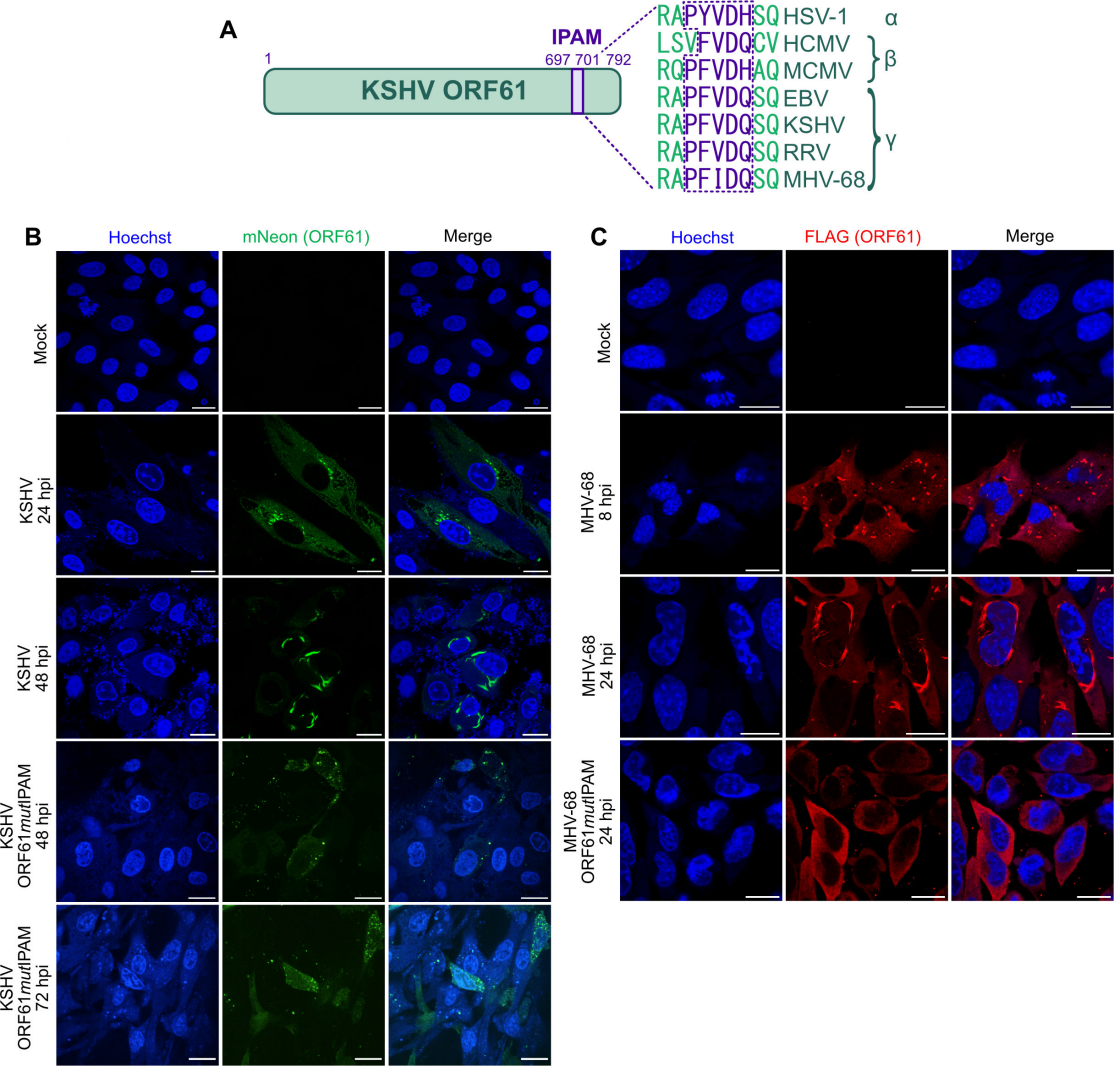
Rhadinovirus R1 proteins accumulate in elongated cytoplasmic condensates. (**A**) Schematic representation of KSHV ORF61 containing the Induced Protein Aggregation Motif (IPAM) and an alignment of the IPAM sequences within selected herpesvirus R1 homologs, including the γ-herpesviruses EBV, KSHV, RRV (rhesus rhadinovirus), and MHV-68. Confocal microscopy images of (**B**) ARPE-19 cells infected with KSHV mNeon-ORF61 or mNeon-ORF61*mut*IPAM and (**C**) 10.1 cells infected with MHV-68 ORF61- or ORF61*mut*IPAM-FLAG. Cells were fixed at different times post-infection, and nuclei were counterstained with Hoechst 33342. 10.1 cells were immunostained with an anti-FLAG antibody. Fluorescence images were acquired by cLSM. Scale bar, 20 µm.

We have previously shown that the R1 proteins of MCMV and HSV-1 form aggregates in the cytoplasm of infected cells to sequester cellular target proteins. This process depends on an IPAM present in the C-terminal part of these proteins (8). As this motif is also conserved in R1 proteins of both human and non-human γ-herpesviruses (Fig. 1A), we wanted to determine whether the R1 proteins of two rhadinoviruses, KSHV and MHV-68, also form cytoplasmic condensates and whether the IPAM is involved in this process. To facilitate these studies in the context of lytic viral infection, we used a KSHV bacterial artificial chromosome (BAC) clone that was modified to enforce the expression of the viral replication and transcription activator (RTA) protein (26, 27). Virus reconstituted from the KSHV_Lyt_ BAC replicates to usable titers in human retinal pigment epithelial cells (RPE-1 and ARPE-19 cells) (26). This KSHV_Lyt_ BAC served as the backbone to construct a recombinant KSHV encoding a green fluorescent protein (mNeonGreen) fused to the viral R1, ORF61. To determine the role of the IPAM, we mutated two crucial amino acids of the motif (DQ to AA) to generate ORF61*mut*IPAM. Human ARPE-19 cells were infected with recombinant KSHVs and analyzed by confocal laser scanning microscopy (cLSM). Throughout the infection, we observed dynamic changes in the mNeon-tagged ORF61 protein. The ORF61 protein accumulated in cytoplasmic condensates that increased considerably in size and acquired an elongated morphology as the infection proceeded (Fig. 1B). Very similar elongated ORF61 condensates were also detected in KSHV-infected telomerase-immortalized microvascular endothelial (TIME) cells, indicating that this phenotype is not restricted to epithelial cells (Fig. S1A). In contrast, mNeon-tagged ORF61*mut*IPAM did not form the same elongated condensates. Although small dot-like structures were occasionally detected, they did not grow in the course of infection, and the ORF61*mut*IPAM protein was generally more dispersed throughout the cytoplasm even at very late times (72 h) post-infection (Fig. 1B). This suggests that an intact IPAM is required for the formation of elongated ORF61 condensates.

To test whether the peculiar morphology of the ORF61 condensates is conserved in other γ2-herpesviruses, we analyzed ORF61 of MHV-68, a related rodent rhadinovirus. By BAC recombineering, we generated a recombinant MHV-68 expressing mNeon-tagged ORF61, analogous to the KSHV mutants described above. In mouse fibroblasts infected with the mNeon-tagged ORF61 virus, dot-like structures became visible around 6 h post-infection (hpi). They grew over time and acquired an elongated morphology (Fig. S1B), structurally resembling KSHV ORF61 condensates (Fig. 1B).

To ensure that the striking morphology of the KSHV and MHV-68 ORF61 condensates was not an artifact caused by the mNeonGreen tag, we constructed a recombinant MHV-68 expressing ORF61 fused C-terminally with a small FLAG epitope tag. FLAG-tagged ORF61 was detected within MHV-68-infected cells by immunofluorescence staining at different times post-infection. As shown in Fig. 1C, FLAG-tagged ORF61 formed condensates similar to those observed with mNeon-tagged ORF61 (Fig. S1B). Disruption of the IPAM by two alanine substitutions (ORF61*mut*IPAM) resulted in a loss of condensate formation and a dispersed cytoplasmic distribution of ORF61, even at late times post-infection (Fig. 1C), confirming that the IPAM is required for condensate formation.

### Rhadinovirus R1 proteins become insoluble during infection

**Fig 2:**
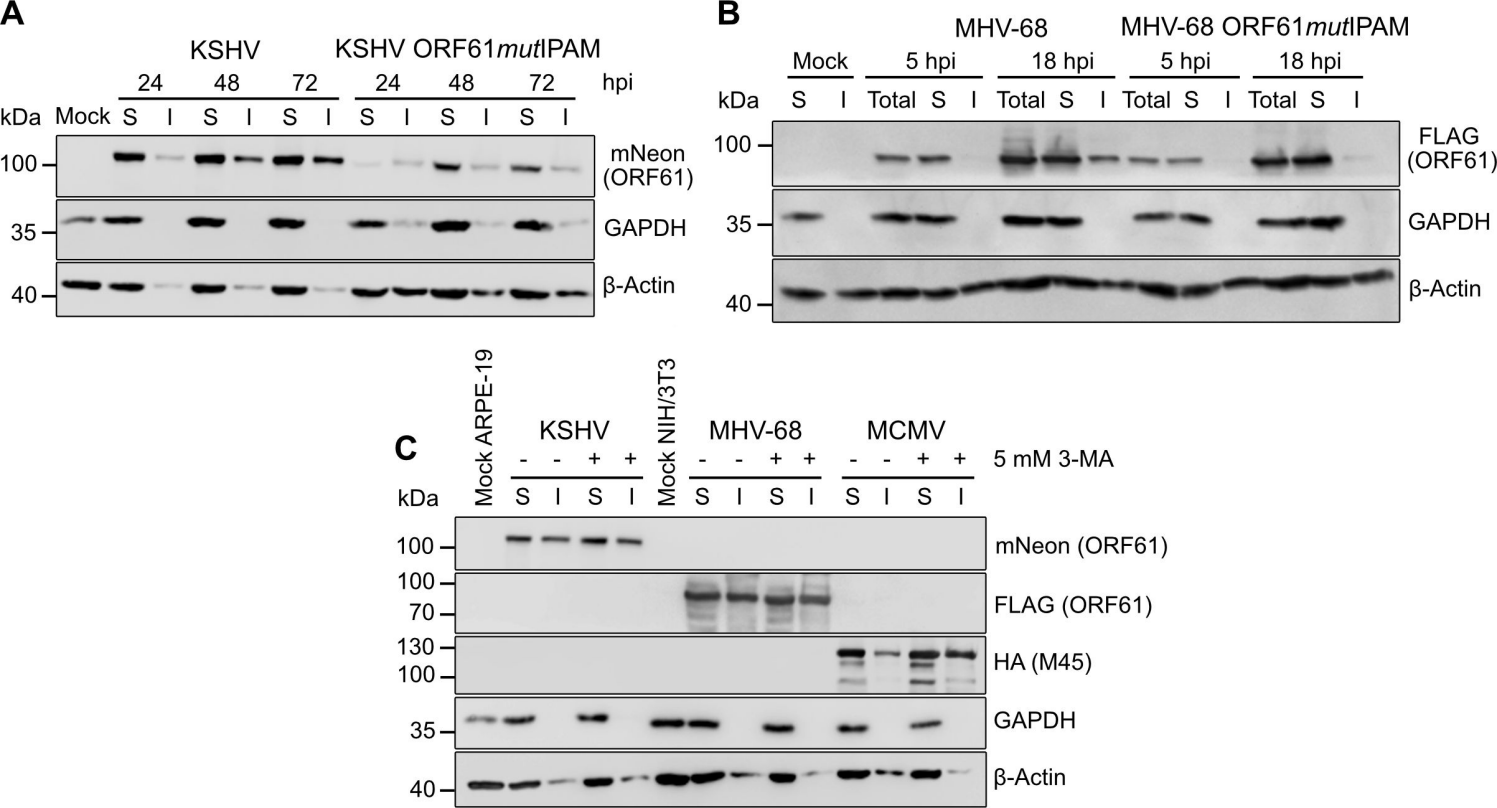
Rhadinovirus R1 proteins become insoluble during infection. (**A**) ARPE-19 cells were infected with KSHV mNeon-ORF61 or mNeon-ORF61*mut*IPAM (MOI 0.1) and harvested at different times post-infection. The detergent-soluble (S) and insoluble (I) fractions were analyzed by immunoblot. GAPDH was used as a fractionation control and β-Actin as a loading control. (**B**) Murine 10.1 fibroblasts were infected with MHV-68 ORF61-FLAG or ORF61*mut*IPAM-FLAG (MOI 2) and harvested at different times post-infection. Total cell lysates and the detergent-soluble (S) and insoluble (I) fractions were analyzed by immunoblot. (**C**) ARPE-19 cells were infected with KSHV mNeon-ORF61 at MOI 0.1 and NIH/3T3 fibroblasts with either MHV-68 ORF61-FLAG or MCMV M45-HA at MOI 5. At 6 hpi, cells were treated with 5 mM 3-methyladenine (3-MA) until lysate collection to inhibit autophagy or left untreated. Cell lysates were collected at 24 hpi and separated into soluble (S) and insoluble (I) fractions.

As shown in Fig. 1, KSHV and MHV-68 R1 proteins undergo dynamic structural changes during infection, and this reorganization requires an intact IPAM. To assess the impact of these changes on the solubility of the R1 proteins, we infected ARPE-19 cells with KSHV expressing mNeon-tagged ORF61 (Fig. 2A) and mouse fibroblasts with MHV-68 expressing FLAG-tagged ORF61 (Fig. 2B), separated the infected cell lysates into soluble and insoluble fractions by centrifugation, and detected ORF61 by immunoblot. Whereas the ORF61 protein of both viruses was detected predominantly in the soluble fraction at early times post-infection, it gradually accumulated in the insoluble fraction at later times. Such a change in solubility was not observed with ORF61*mut*IPAM, which remained soluble throughout the infection, further underpinning the importance of the IPAM for the structural properties of ORF61. Although a small amount of insoluble KSHV ORF61*mut*IPAM was detected, the levels were markedly lower compared with ORF61 and remained constant over time without a gradual increase.

In a previous study, we showed that MCMV and HSV-1 R1 protein aggregates are degraded by selective autophagy (8). To test if the insoluble ORF61 aggregates are also degraded by autophagy, we infected ARPE-19 cells with KSHV mNeon-ORF61 and mouse fibroblasts with MHV-68 ORF61-FLAG in the presence or absence of the autophagy inhibitor 3-methyladenine (3-MA). Soluble and insoluble ORF61 was detected by immunoblot (Fig. 2C). Whereas increased levels of the MCMV R1 protein, M45, were detected in the insoluble fraction upon 3-MA treatment (positive control), such an increase was not detected with KSHV or MHV-68 ORF61, suggesting that autophagy does not play a major role in the turnover of rhadinovirus ORF61 aggregates.

### Rhadinovirus ORF61 protein condensates are solid filamentous aggregates

**Fig 3:**
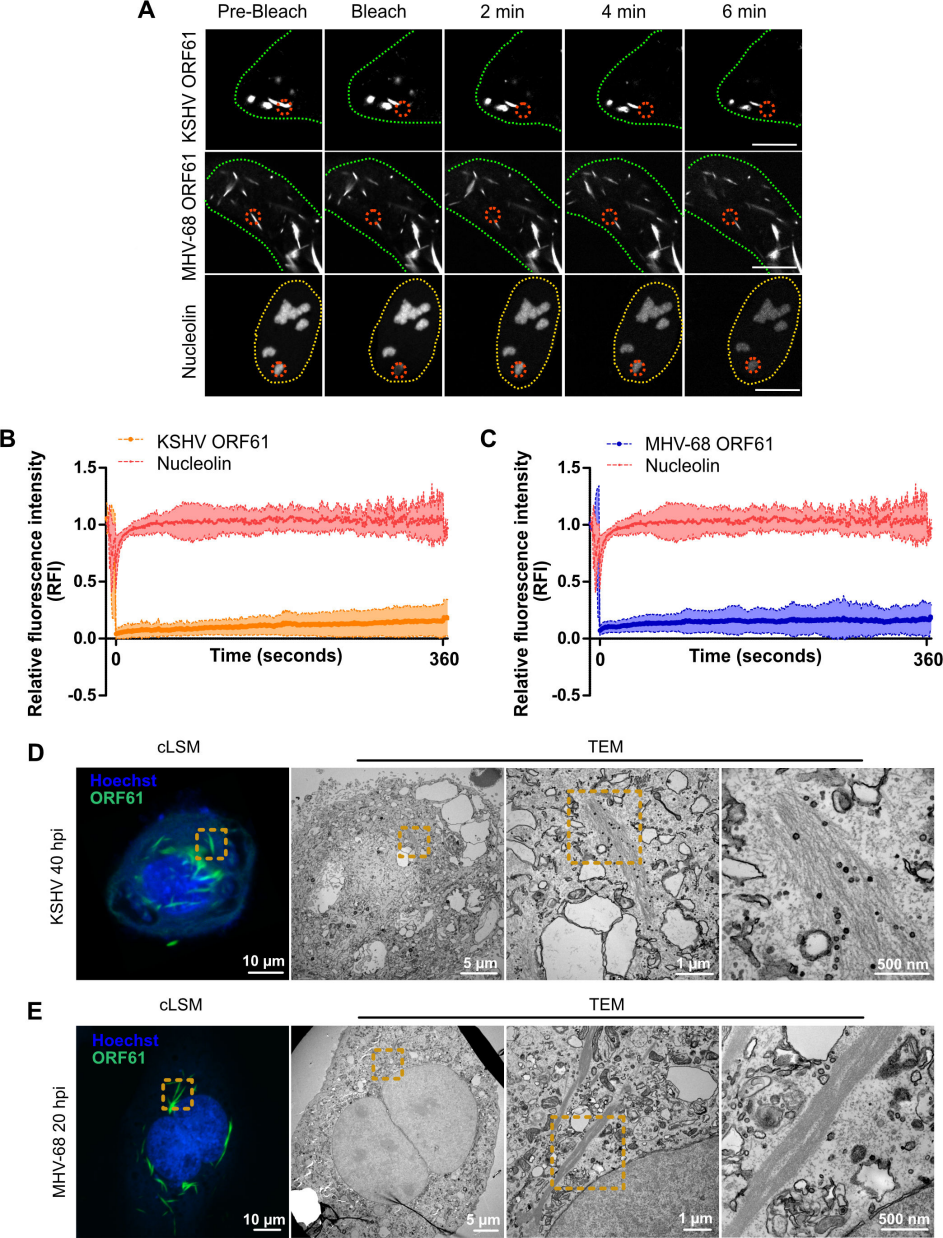
Rhadinovirus R1 protein condensates are solid aggregates composed of filamentous bundles. (**A**) Fluorescence recovery after photobleaching (FRAP) analysis of mCherry-tagged R1 proteins. KSHV and MHV-68 ORF61-pmCherry were expressed in U2OS cells by plasmid transfection. mCherry-positive structures were half-bleached, and fluorescence recovery was recorded at two frames per second for 6 min. pmCherry-Nucleolin was used as a control. Scale bar, 10 µm. (B and C) Relative fluorescence intensity (RFI) of bleached areas over time in at least 10 different cells per sample. The shaded areas are the mean values ± SEM. For correlative light and electron microscopy (CLEM) of ORF61 aggregates, (**D**) ARPE-19 cells were infected with KSHV mNeon-ORF61, and (**E**) MEF cells with MHV-68 ORF61-mNeon. At 40 hpi (KSHV) or 20 hpi (MHV-68), the cells were fixed, and nuclei were stained with Hoechst 33342. Fluorescent Z-stacks were acquired by cLSM. Ultrathin 50 nm serial sections of the samples were prepared and imaged by transmission electron microscopy (TEM). Representative overview images and magnified views of the indicated areas are shown.

To further characterize the elongated cytoplasmic condensates formed by KSHV and MHV-68 ORF61, we expressed them as mCherry-tagged fusion proteins in cells and measured fluorescence recovery after photobleaching (FRAP) (Fig. 3A). Unlike mCherry-tagged Nucleolin, which forms liquid condensates that rapidly recover after photobleaching (8, 28), the mCherry-tagged ORF61 protein condensates showed no recovery after photobleaching (Fig. 3B and C), suggesting that ORF61 proteins form solid protein aggregates. We then used correlative light and electron microscopy (CLEM) to visualize aggregates in cells infected with KSHV or MHV-68 expressing mNeon-tagged ORF61. In both cases, the elongated aggregates consisted of filamentous bundles. The filaments in KSHV-infected cells were rather loosely packed (Fig. 3D), whereas the ones in MHV-68-infected cells formed more compact bundles (Fig. 3E). Due to their resemblance to actin filaments, we tested whether ORF61 aggregates contain F-actin by using rhodamine-phalloidin staining. However, F-actin was not detected within ORF61 aggregates (Fig. S2A). As protein aggregates within aggresomes can be surrounded by vimentin (29), an intermediate filament protein that is expressed in mesenchymal cells such as fibroblasts (30), we infected human foreskin fibroblasts (HFF) with KSHV mNeon-ORF61 and stained for vimentin by indirect immunofluorescence. However, vimentin was not detected, neither within nor surrounding ORF61 aggregates (Fig. S2B). These data suggested that the rhadinovirus ORF61 proteins form elongated aggregates consisting of filamentous bundles that do not contain F-actin or vimentin.

### APOBEC3B localizes to KSHV ORF61 aggregates in transfected cells

**Fig 4:**
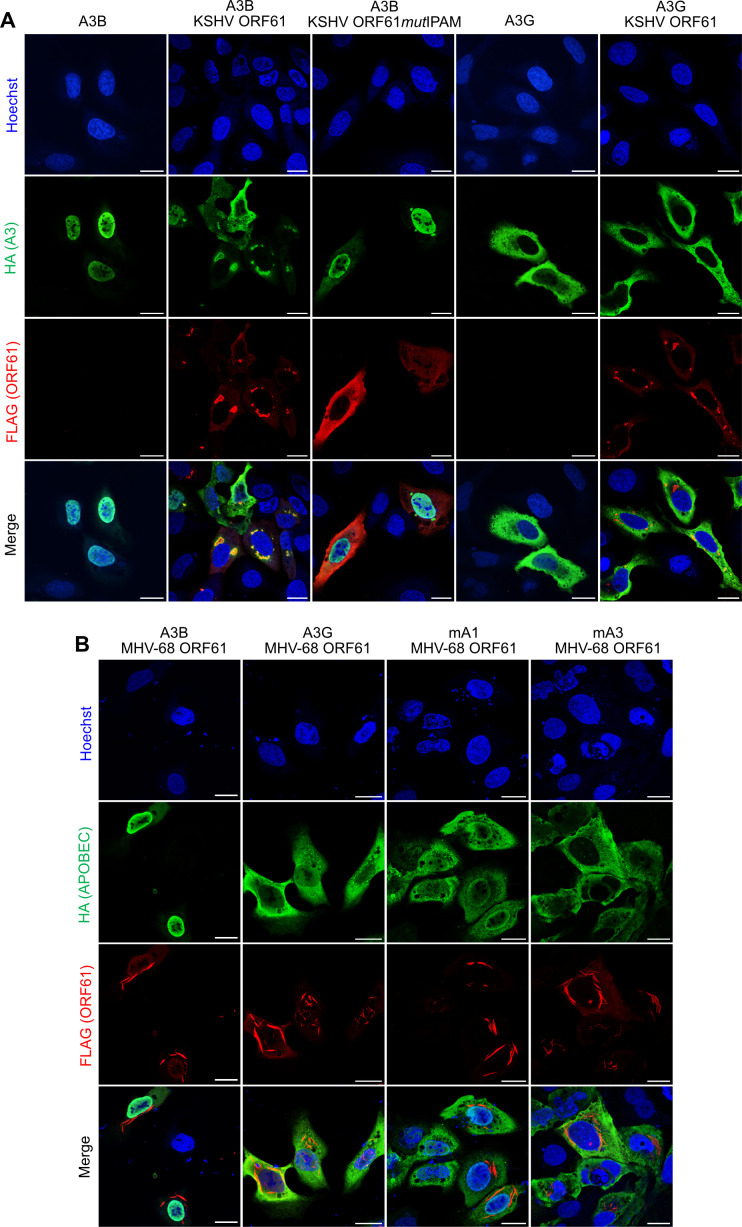
KSHV ORF61 relocalizes APOBEC3B to aggregates, whereas MHV-68 ORF61 does not relocalize human or mouse APOBECs. U2OS cells were transfected with plasmids expressing (**A**) A3B-HA or A3G-HA and KSHV ORF61-FLAG or ORF61*mut*IPAM-FLAG, (**B**) A3B-HA, A3G-HA, mA1-HA, or mA3-HA and MHV-68 ORF61-FLAG. Cells were fixed, immunostained with anti-HA and anti-FLAG antibodies, and counterstained with Hoechst 33342. Fluorescence images were acquired by cLSM. Scale bar, 20 µm.

Previous studies have demonstrated that the EBV and HSV-1 R1 proteins, BORF2 and ICP6, interact with the cellular cytosine deaminase APOBEC3B (A3B) and relocalize it from the nucleus to the cytoplasm to protect the replicating viral DNA from its mutagenic effect (14, 16). Therefore, we tested whether the KSHV ORF61 protein also interacts with and relocalizes A3B to filamentous aggregates and whether this process is IPAM-dependent. To test for the specificity of a potential interaction, we included A3G, a cytoplasmic A3 enzyme (31), as a control. U2OS cells were transfected with plasmids expressing FLAG-tagged ORF61 or ORF61*mut*IPAM and HA-tagged A3B or A3G, and the subcellular localization of the proteins was determined by indirect immunofluorescence. As shown in Fig. 4A, A3B colocalized with elongated ORF61 aggregates in the cytoplasm, but A3G did not. The colocalization was lost when the IPAM was mutated. As MHV-68 ORF61 forms similar elongated condensates as its KSHV homolog (Fig. 1C), we tested whether MHV-68 ORF61 could also relocalize A3B to cytoplasmic aggregates in transfected cells. However, this was not the case (Fig. 4B), suggesting that the ability to interact with human A3B is not conserved in MHV-68 ORF61. This finding was not entirely unexpected as rodent cells, the natural host cells of MHV-68, lack APOBEC3B and express a single cytoplasmic APOBEC3 protein, in contrast to the seven A3 proteins found in humans and related primates (19). Hence, we tested whether the MHV-68 ORF61 protein could sequester murine APOBEC3 (mA3). However, neither mA3 nor the more distantly related murine APOBEC1 (mA1) was relocalized by MHV-68 ORF61 in transfected cells (Fig. 4B), suggesting that MHV-68 ORF61 does not sequester these proteins in cytoplasmic aggregates.

Herpesviral R1 proteins are known to interact with other cellular proteins besides A3B: HSV-1 ICP6 binds RIPK1, and MCMV M45 binds both RIPK1 and NEMO to prevent necroptosis and NF-κB signaling (5–8, 32). To test if rhadinovirus R1 proteins share the same cellular targets, we co-expressed KSHV and MHV-68 ORF61-HA with either FLAG-tagged human or murine RIPK1 or NEMO and precipitated the R1 proteins using an anti-HA antibody. Whereas RIPK1 and NEMO co-precipitate with MCMV M45, they did not co-precipitate with KSHV and MHV-68 R1 proteins, indicating that these interactions are not conserved in rhadinoviruses (Fig. S3).

### KSHV ORF61 requires an intact IPAM for A3B binding and relocalization during infection

**Fig 5:**
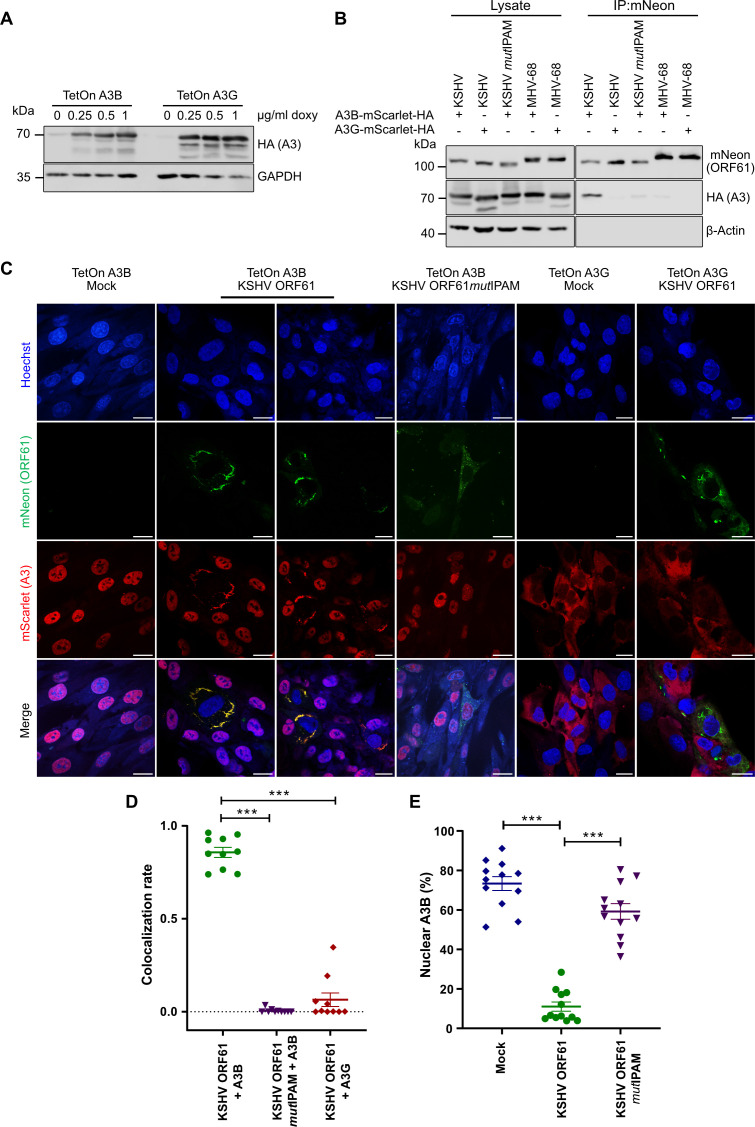
KSHV ORF61 binds and relocalizes A3B in an IPAM-dependent manner in infected cells. (**A**) The Tet-inducible expression of mScarlet-HA-tagged A3B or A3G in stably transduced RPE-1 cells was verified by treating the cells with increasing concentrations of doxycycline for 24 h and detecting the A3 proteins by immunoblot. (**B**) TetOn A3B and A3G RPE-1 cells were infected with either KSHV mNeon-ORF61, KSHV mNeon-ORF61*mut*IPAM (MOI 0.1), or MHV-68 ORF61-mNeon (MOI 5). A3 expression was induced 16 hpi with 1 µg/mL doxycycline, and cell lysates were used for mNeon pulldown at 40 hpi. Co-precipitating proteins were detected by immunoblot. (**C**) TetOn A3B and A3G RPE-1 cells infected with KSHV mNeon-ORF61 or KSHV mNeon-ORF61*mut*IPAM. A3 expression was induced 16 hpi with 1 µg/mL doxycycline. At 48 hpi, cells were fixed, nuclei were counterstained with Hoechst 33342, and fluorescence images were acquired by cLSM. Scale bar, 20 µm. (**D**) Colocalization was quantified by calculating Pearson’s correlation coefficient for ORF61 and A3 signals using Z-stacks acquired for each condition (*n* = 10). Mean values ± SEM are shown. Significance was calculated using an unpaired Student’s *t*-test. ***, *P* < 0.001. (**E**) The percentage of nuclear A3B fluorescence intensity was determined by dividing the nuclear by the total A3B intensity for each infected cell (*n* = 12). Mean values ± SEM are shown. Significance was calculated using an unpaired Student’s *t*-test. ***, *P* < 0.001.

Having observed that KSHV ORF61 colocalizes with A3B in transfected cells, whereas MHV-68 ORF61 does not, we wanted to verify our findings in the context of infection. As A3B-specific antibodies suitable for immunofluorescence were not commercially available, we generated human RPE-1 cells expressing either A3B or A3G in a doxycycline-inducible fashion. A3B and A3G were fused with the red fluorescent protein mScarlet and an HA tag for detection by fluorescence microscopy and immunoblot, respectively (Fig. 5A). These cells were infected with KSHV and MHV-68 viruses expressing mNeon-tagged ORF61 or ORF61*mut*IPAM. A3B or A3G expression was induced with doxycycline. The interaction of ORF61 with APOBEC3 proteins was analyzed by immunoprecipitation and immunoblot analysis. As shown in Fig. 5B, A3B co-precipitated with the KSHV ORF61 protein but not with KSHV ORF61*mut*IPAM or MHV-68 ORF61, and A3G did not co-precipitate with either of the viral ORF61 proteins.

To corroborate these results and verify that KSHV ORF61 relocalizes and sequesters nuclear A3B in the cytoplasm during infection, we infected A3B- and A3G-mScarlet-expressing cells with KSHV mNeon-ORF61 and mNeon-ORF61*mut*IPAM (Fig. 5C). Indeed, A3B-mScarlet was relocalized from the nucleus to the cytoplasm where it colocalized with the filamentous cytoplasmic aggregates formed by ORF61. In contrast, the localization of cytoplasmic A3G was not affected, and A3G did not accumulate in ORF61 aggregates (Fig. 5D and E). The ORF61*mut*IPAM virus was unable to relocalize A3B (Fig. 5D and E). Taken together, these results indicated that KSHV ORF61 relocalizes and sequesters nuclear A3B in cytoplasmic aggregates during lytic infection and that an intact IPAM is required for relocalization and sequestration.

### KSHV ORF61 is required for efficient replication and protection from A3B-mediated genome editing

**Fig 6:**
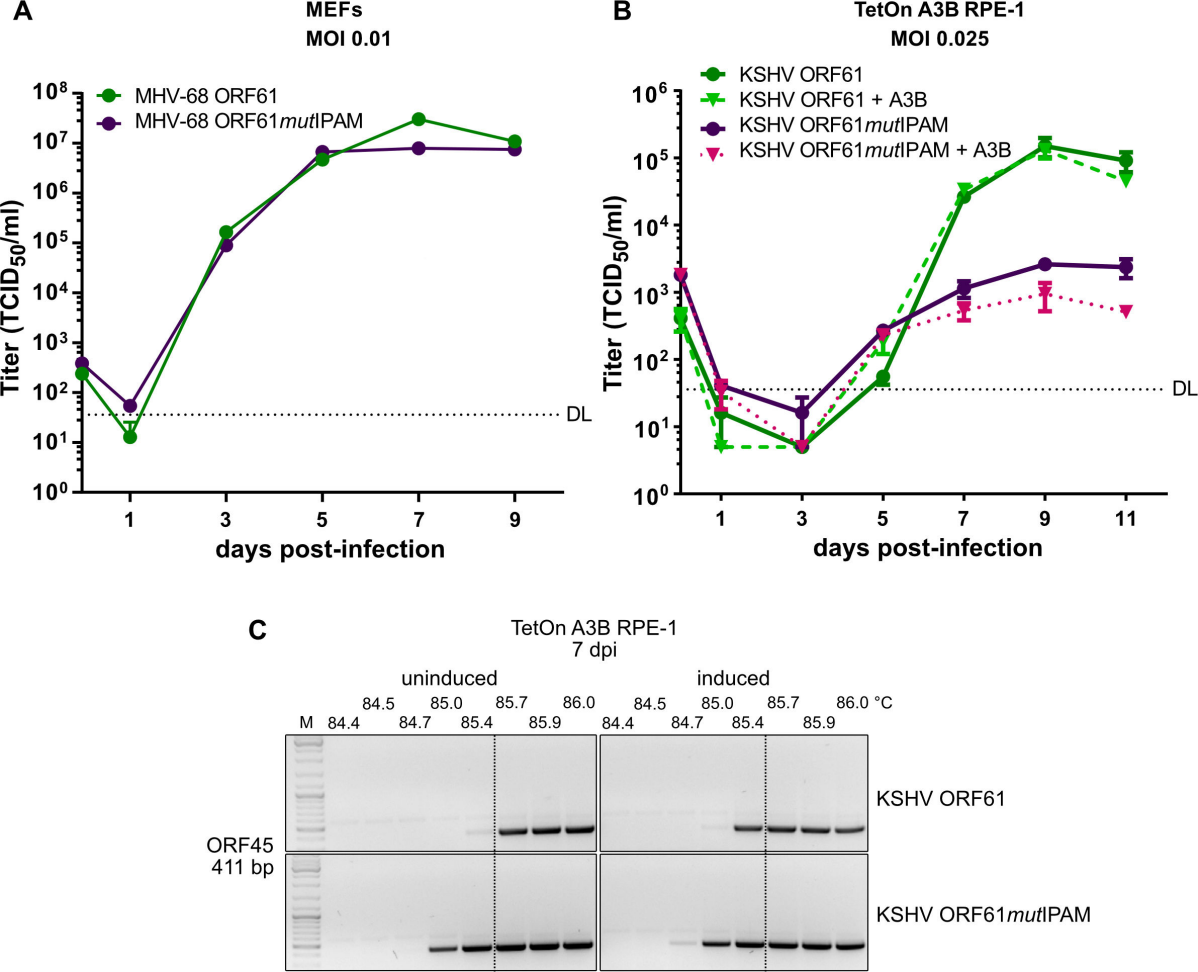
Requirement of the KSHV ORF61 IPAM for efficient replication and protection from A3B-mediated genome editing. (**A**) Multistep MHV-68 replication kinetics. MEF cells were infected with MHV-68 ORF61-FLAG or ORF61*mut*IPAM-FLAG at MOI 0.01. Viral titers in the supernatants were determined. Mean values ± SEM of triplicates are shown. DL, detection limit. (**B**) Multistep KSHV replication kinetics. TetOn A3B RPE-1 cells were infected with either KSHV mNeon-ORF61 or mNeon-ORF61*mut*IPAM at MOI 0.025. At 4 hpi, the cells were treated with 1 µg/mL doxycycline to induce A3B expression or left untreated. Viral titers were determined in the supernatants. Mean values ± SEM of triplicates are shown. DL, detection limit. (**C**) Differential DNA denaturation PCR (3D-PCR) of KSHV mNeon-ORF61 and mNeon-ORF61*mut*IPAM with (induced) or without (uninduced) A3B overexpression in TetOn A3B RPE-1 cells. DNA extracted from supernatants at 7 dpi was used to PCR-amplify a fragment of the viral ORF45. A PCR with a denaturation temperature gradient was used to determine the lowest denaturation temperature that allowed amplification. The dotted lines indicate the lowest denaturation temperature for the ORF45 fragment of KSHV mNeon-ORF61 without A3B induction. M, DNA size marker.

Our previous findings demonstrated that the rhadinovirus ORF61 proteins form elongated filamentous cytoplasmic aggregates in an IPAM-dependent manner. In the case of KSHV, the ORF61 protein binds to A3B and relocalizes it to these aggregates. However, the importance of aggregate formation and A3B sequestration for viral replication had not been considered. To test this, we analyzed the replication kinetics of MHV-68 and KSHV mutants carrying an intact or mutant ORF61. As shown in Fig. 6A, MHV-68 ORF61*mut*IPAM replicated with very similar kinetics in MEF cells, suggesting that the ability to form cytoplasmic ORF61 aggregates had no major impact on MHV-68 replication in these cells. In contrast, KSHV ORF61*mut*IPAM had a substantial replication defect in TetOn A3B RPE-1 cells compared to the control virus without the IPAM mutation (Fig. 6B). When A3B overexpression was induced with doxycycline, titers were even more reduced. These results suggested that A3B sequestration might be important for viral replication and, at least in part, responsible for the growth attenuation.

Next, we wanted to determine whether the ORF61*mut*IPAM virus was more susceptible to A3B-mediated genome deamination. To test this, we applied differential DNA denaturation PCR (3D-PCR), an established method to detect APOBEC3-induced hypermutations. It allows differential amplification of template DNA based on its GC content by making use of the lower temperature required to denature AT-rich sequences (33). As APOBEC3 enzymes deaminate cytosine residues to uracil, which leads to a G-to-A hypermutation on the complementary strand (21), the GC content of edited sequences is reduced, allowing their amplification by 3D-PCR at lower denaturation temperatures.

TetOn A3B RPE-1 cells were infected with KSHV mNeon-ORF61 or ORF61*mut*IPAM with or without doxycycline induction. Viral DNA was extracted from supernatants on day 7 post-infection and used to PCR-amplify a 577 bp fragment of the viral immediate-early gene ORF45, which contains several 5′-TC-dinucleotides suitable for A3B-mediated deamination (34). Equal amounts of the PCR products were then used as a template for a second-round PCR amplification of a smaller 411 bp fragment using a denaturation temperature gradient from 84.4 to 86°C. As shown in Fig. 6C, the ORF45 fragment of KSHV mNeon-ORF61*mut*IPAM was amplified at lower denaturation temperatures compared with that of the control virus (left panel). Doxycycline-induced A3B overexpression led to a shift in the minimal denaturation temperature for both viruses (right panel), suggesting that genome deamination by overexpressed A3B was incompletely counteracted by ORF61 in infected RPE-1 cells. These results confirmed that ORF61-mediated sequestration of A3B to filamentous cytoplasmic aggregates serves to protect the viral genome from A3B-mediated deamination.

## DISCUSSION

In this study, we show that the R1 proteins of two rhadinoviruses, KSHV and MHV-68, form elongated condensates in virus-infected cells. These condensates have properties of solid aggregates: they are detergent-insoluble (Fig. 2), do not recover fluorescence after photobleaching (Fig. 3A through C), and consist of filamentous bundles when analyzed by CLEM (Fig. 3D and E). This shape is unusual and strikingly different from the previously described R1 aggregates formed by the MCMV and HSV-1 R1 proteins. M45 and ICP6 aggregates appear to be globular when analyzed by fluorescence microscopy, and the M45 aggregates have an amorphous bulky appearance when visualized by CLEM (8). Although the structure of the purified EBV BORF2 protein has been determined by cryo-EM (15), the morphology of the BORF2 condensates in EBV-infected cells has not been analyzed in further detail. The reason for the morphological differences among the viral R1 aggregates is unknown. However, the viral R1 proteins are known to self-interact (8, 15) and, thus, have the potential to form higher-order oligomers. Further cryo-EM analyses of R1 aggregates and structural predictions (e.g., with AlphaFold) will likely clarify how some viral R1 proteins, such as the rhadinovirus ORF61 proteins, form filamentous structures, whereas others form aggregates of different shapes.

Viral proteins forming filamentous structures are generally not unheard of. For instance, Rift Valley fever virus (RVFV), an RNA virus of the order Bunyavirales, causes the formation of large nuclear filament bundles mainly composed of the viral nonstructural small (NSs) protein (35). EM analysis of these nuclear structures revealed 0.5 µm wide bundles consisting of thinner parallel fibrils (36), strikingly resembling the structural organization we observe for the cytoplasmic ORF61 aggregates (Fig. 3D and E). Interestingly, RVFV NSs filaments have also been proposed to serve a sequestration function. Subunits of transcription factor II H (TFIIH), which is required for RNA Pol II initiation, as well as a histone deacetylase complex regulating interferon-β production, are present in these nuclear viral filaments (37, 38).

Unlike MCMV and HSV-1 R1 aggregates, which are targeted to autophagosomes for subsequent lysosomal degradation (6, 8), rhadinovirus R1 aggregates do not seem to undergo degradation by autophagy (Fig. 2C). Maybe the filamentous ORF61 aggregates do not bind or attract cargo receptors to stimulate autophagy as M45 and ICP6 do. Another possible explanation might be their size and shape, which render them resistant to degradation by autophagy.

Although R1 proteins of different herpesviruses differ in terms of morphological features and cellular processing, they share the property of aggregation to sequester distinct, albeit partially overlapping, sets of cellular target proteins. MCMV M45 sequesters NEMO and RIPK1 (8); HSV-1 ICP6 sequesters RIPK1 and A3B (8, 16); and EBV BORF2 and KSHV ORF61 sequester A3B (14 and this study). All these viral proteins contain an IPAM (Fig. 1A). Mutation of this motif abrogates both aggregate formation and sequestration of the respective cellular target proteins (8, 9 and this study), indicating a crucial role of the IPAM in this process. Structural studies on EBV BORF2 have identified specific N-terminal amino acids that are also involved in self-interaction and oligomerization, but these residues are only partially conserved in other herpesvirus R1 proteins (15).

In the case of KSHV, an intact IPAM is required for efficient viral replication, as we show here. Although the temporal kinetics of ORF61*mut*IPAM virus replication are similar to those of the parental virus, the peak titers are considerably reduced (Fig. 6B). Interestingly, A3B overexpression further restricts IPAM mutant replication and increases the mutant’s susceptibility to A3B-mediated deamination (Fig. 6B and C). These findings suggest that the failure to form ORF61 aggregates to remove the mutagenic A3B from the nucleus or to block its enzymatic function is at least partially responsible for the observed replication defect of KSHV ORF61*mut*IPAM. However, as the IPAM lies in the vicinity of the R1 catalytic residues, an impaired RNR enzymatic function cannot be excluded. A KSHV ORF61 mutant unable to sequester A3B while retaining all other protein functions would be needed to distinguish between A3B-dependent and -independent effects. Furthermore, it would be interesting to test whether KSHV genome deamination and reduced replication of the ORF61 IPAM mutant could be reversed or alleviated in A3B knockout cells.

In contrast to the R1 proteins of MCMV, HSV-1, EBV, KSHV, and MHV-68, the HCMV R1 protein, UL45, does not contain a complete IPAM (Fig. 1A). It does not form cytoplasmic protein condensates, nor does it interact with A3A or A3B in co-immunoprecipitation experiments (39). Nonetheless, HCMV does induce relocalization of A3B from the nucleus to the cytoplasm, but this process occurs through an unknown, UL45-independent mechanism (39).

The ORF61 protein of MHV-68 forms filamentous cytoplasmic aggregates in an IPAM-dependent manner, similar to KSHV ORF61 (Fig. 1C). However, unlike its KSHV homolog, MHV-68 ORF61 does not relocalize and sequester A3 proteins (Fig. 4B and 5B), and in contrast to the KSHV ORF61 IPAM mutant, the MHV-68 IPAM mutant shows no obvious replication defect in fibroblasts (Fig. 6A). Previous studies showed that the presence or absence of murine A3 did not influence MHV-68 replication or pathogenesis *in vivo* (40, 41), suggesting that murine A3 is not a restriction factor for this virus. However, it seems highly unlikely that MHV-68 forms ORF61 protein aggregates without using them to sequester cellular factors involved in virus restriction or innate immunity. A previous study presented data suggesting that MHV-68 ORF61 induces an altered morphology of the antiviral promyelocytic leukemia nuclear bodies (42). However, the cellular interactors of MHV-68 ORF61 remain elusive. Moreover, the R1 protein aggregates of other herpesviruses may contain additional cellular target proteins besides the known ones. Hence, it should be worthwhile to determine the proteome of purified R1 aggregates in future studies.

## MATERIALS AND METHODS

### Cells

Human ARPE-19 retinal pigment epithelial cells (ATCC CRL-2302) were maintained in Dulbecco’s modified Eagle medium (DMEM)/F-12 GlutaMAX (Gibco) supplemented with 10% fetal calf serum (FCS), 15 mM HEPES (Gibco), 1 mM sodium pyruvate (Gibco), 100 U penicillin, and 100 µg/mL streptomycin (P/S) (Sigma). 10.1 (43) and NIH/3T3 (ATCC CRL-1658) mouse fibroblasts, MEF cells (Riken BRC RCB2710), HEK-293A cells (Invitrogen), U2OS osteosarcoma cells (ATCC HTB-96), life-extended HFF (44), and hTERT-RPE-1 human retinal pigment epithelial cells (ATCC CRL-4000) were maintained in DMEM (PAN-Biotech) supplemented with 10% FCS, 15 mM HEPES, and P/S. hTERT-immortalized human microvascular endothelial (TIME) cells (45) were maintained in EBM-2 Basal Medium supplemented with EGM-2 MV Microvascular Endothelial SingleQuots (Lonza) on gelatin-coated cell culture dishes. All cells were grown at 37°C and 5% CO_2_.

### Viruses

All recombinant viruses were generated by en passant BAC mutagenesis (46). The KSHV_Lyt_ BAC (26, 47, 48), which is based on the JSC-1-derived KSHV BAC16 (49), was used as the backbone for recombinant KSHVs. For KSHV mNeon-ORF61, the EF1α-eGFP-IRES-HygroR-BGH cassette within the BAC vector was deleted and replaced by a zeocin resistance marker. Then, the mNeonGreen sequence followed by a linker sequence was inserted at the 5' end of ORF61. For KSHV mNeon-ORF61*mut*IPAM, the IPAM within ORF61 (aa positions 697-701) was mutated by two alanine substitutions (PFVDQ to PFVAA). Recombinant viruses were grown and titrated on ARPE-19 or RPE-1 cells. Titers were determined using the median tissue culture infective dose (TCID_50_) method (50) with plaque formation as a readout. In our experiments, an MOI of 0.1 (TCID_50_/cell) was sufficient to infect almost all the cells. Hence, plaque-forming units are an underestimate of KSHV_Lyt_ infectivity, and the KSHV_Lyt_ titers are not directly comparable with those of naturally lytic replicating viruses.

The MHV-68 BAC pHA3 (51) was used to construct recombinant MHV-68 strains. For MHV-68 ORF61-FLAG, a 3xFLAG-tag sequence preceded by a short linker was introduced to the 3' end of ORF61. For MHV-68 ORF61*mut*IPAM-FLAG, the IPAM within ORF61 (aa positions 681-685) was mutated by two alanine substitutions (PFIDQ to PFIAA). For MHV-68 ORF61-mNeon, the eGFP expression cassette within the BAC vector was deleted and replaced by a zeocin resistance marker. Then, the mNeonGreen sequence preceded by a linker sequence was inserted at the 3' end of ORF61. Recombinant MHV-68 strains and MCMV M45-HA (52) were grown and titrated on MEF or 10.1 cells.

The integrity of all recombinant BACs was verified by restriction fragment length pattern analysis and sequencing of the modified regions. The genome sequences of ORF61*mut*IPAM viruses, which are derivatives of the corresponding tagged ORF61 viruses, were additionally subjected to Oxford Nanopore sequencing. No deviations from the expected sequence were detected for KSHV ORF61*mut*IPAM. In MHV-68 ORF61*mut*IPAM, two non-synonymous C-to-A substitutions were detected at positions 100,512 and 100,812 within the internal repeat region (M10 locus). These positions do not overlap with the viral oriLyt, and the remainder of M10 does not impact viral replication *in vitro* or *in vivo* (53). Hence, these two substitutions were considered irrelevant.

### Antibodies

Antibodies recognizing the following antigens were used: mNeonGreen (32F6, Proteintech), GAPDH (14C10, Cell Signaling), β-Actin (AC-74, Sigma), FLAG (M2, Sigma), HA (3F10, Roche), HA (H6908, Sigma), and vimentin (RV202, Santa Cruz). Secondary antibodies conjugated to horseradish peroxidase (HRP) were purchased from DakoCytomation or Jackson ImmunoResearch. Secondary antibodies conjugated to Alexa Fluor 488, 555, or 647 were purchased from Thermo Fisher Scientific.

### Plasmids and transfection

Plasmids pcDNA3-A3B-HA (54) and pcDNA3-A3G-HA (55) were kindly provided by Bryan Cullen (Duke University Medical Center, Durham, NC). For the generation of KSHV ORF61-pmCherry, MHV-68 ORF61-pmCherry, pcDNA3-KSHV ORF60-HA, pcDNA3-KSHV ORF61-3xFLAG, pcDNA3-KSHV ORF61-HA, pcDNA3-MHV-68 ORF60-HA, pcDNA3-MHV-68 ORF61-3xFLAG, and pcDNA3-MHV-68 ORF61-HA, the corresponding viral ORF sequences were PCR-amplified either from KSHV BAC16 or MHV-68 BAC pHA3 and inserted between unique restriction sites in pmCherry-N1 (Clontech) or pcDNA3 (Invitrogen). The tag sequences were included with the reverse primers. To generate pcDNA3-KSHV ORF61*mut*IPAM-3xFLAG, the IPAM within ORF61 (aa positions 697-701) was mutated by four alanine substitutions (PFVDQ to PAAAA). pmCherry-Nucleolin (8) and pcDNA3-MCMV M45-HA (5) have been described. pcDNA3.1-hRIPK1-Flag (#112487) and pCMVTAG-NEMO (#11970) were purchased from Addgene. The murine ripk1 cDNA from pMSCVpuro-mRIP1 (5) was subcloned to generate pcDNA3-mRIPK1-Flag. pcDNA3-mA1-3xHA and pcDNA3-mA3-3xHA were generated by Gibson assembly using NEBuilder HiFi DNA Assembly Master Mix (NEB). The murine APOBEC1 and APOBEC3 sequences were PCR-amplified from pCMV-mA1-BE3 (Addgene #113421) and pCMV-mA3-BE3 (Addgene #113419), respectively. For pLIX402-A3B-mScarlet-3xHA and pLIX402-A3G-mScarlet-2xHA, human A3B and A3G sequences were PCR-amplified from the plasmids described above and combined by Gibson assembly with a linker, mScarlet- and HA-tag sequences into the pLIX402 TetOn lentiviral vector (Addgene #41394).

U2OS cells were transfected with 400 ng total DNA for 24 h using GenJet transfection reagent (SignaGen) and HEK-293A cells with 6 µg total DNA for 24 h using polyethylenimine (Sigma).

### Inducible expression of tagged A3B and A3G

Lentiviruses were generated by transfecting HEK-293T cells with pLIX402-A3B-mScarlet-3xHA or pLIX402-A3G-mScarlet-2xHA and standard third-generation packaging plasmids as described (56, 57). RPE-1 cells were transduced with lentiviral vectors in the presence of 5 µg/mL polybrene (Sigma). Cells were selected with 2.5 µg/mL puromycin (Sigma), and single-cell clones were obtained by limiting dilution. A3 expression was induced by adding 1 µg/mL doxycycline (Biomol).

### Cell lysate fractionation

Cells were harvested in a mild lysis buffer (50 mM Tris-HCl pH 7.5, 150 mM NaCl, 1% Nonidet P-40) supplemented with an EDTA-free complete mini protease inhibitor cocktail (Roche). The samples were lysed for 30 min on ice with repeated swirling. Lysates were separated into detergent-soluble and -insoluble fractions by centrifugation (21,130 × *g*, 15 min, 4°C). The soluble supernatant and the insoluble cell pellet were combined with SDS-PAGE sample buffer (125 mM Tris-HCl pH 6.8, 20% glycerol, 4% SDS, 10% β-mercaptoethanol, 0.002% bromophenol blue) and boiled for 10 min at 95°C.

### Immunoprecipitation and immunoblot analysis

TetOn A3B and A3G RPE-1 cells were infected with either KSHV mNeon-ORF61, KSHV mNeon-ORF61*mut*IPAM (MOI 0.1), or MHV-68 ORF61-mNeon (MOI 5) using centrifugal enhancement (1065 × *g*, 30 min). A3B or A3G expression was induced 16 hpi with 1 µg/mL doxycycline. At 40 hpi, cells were harvested in RIPA lysis buffer (50 mM Tris-HCl pH 7.5, 150 mM NaCl, 0.1% SDS, 1% Triton X-100, 1% sodium deoxycholate) supplemented with a protease inhibitor cocktail and 25 U/mL benzonase (Merck). The samples were lysed for 30 min on ice with repeated swirling. Insoluble material was pelleted by centrifugation (17,000 × *g*, 10 min, 4°C) and discarded. After pre-clearing with binding control agarose beads (Proteintech), the supernatants were used for pulldown with mNeonGreen-Trap agarose beads (Proteintech) as per manufacturer’s instructions. After 1 h incubation at 4°C with gentle rotation, the beads were washed once with the lysis buffer and twice with washing buffer (50 mM Tris-HCl pH 7.5, 150 mM NaCl, 10% glycerol). The proteins were eluted by boiling in SDS-PAGE sample buffer. For immunoprecipitation in transfection, HEK-293A cells were lysed 24 h post-transfection for 30 min on ice using a mild lysis buffer (50 mM Tris-HCl pH 7.5, 150 mM NaCl, 1% Nonidet P-40) supplemented with a protease inhibitor cocktail. The insoluble material was pelleted by centrifugation (21,130 × *g*, 15 min, 4°C) and discarded. The pre-cleared supernatant was then used for precipitating HA-tagged R1 proteins using anti-HA antibody (H6908, Sigma) and rProtein A Sepharose Fast Flow resin beads (Cytiva). The precipitates were washed three times with buffer 1 (1 mM Tris pH 7.6, 150 mM NaCl, 2 mM EDTA, 0.2% Nonidet P-40), twice with buffer 2 (1 mM Tris pH 7.6, 500 mM NaCl, 2 mM EDTA, 0.2% Nonidet P-40), once with buffer 3 (10 mM Tris pH 7.6) and then eluted in SDS-PAGE sample buffer by boiling.

Immunoblot analysis was performed according to standard protocols. Briefly, denatured samples were separated by SDS-PAGE and transferred onto a nitrocellulose membrane (Amersham) by semi-dry blotting. Proteins of interest were detected with specific primary antibodies and HRP-conjugated secondary antibodies using enhanced chemiluminescence (ECL) detection reagent (Amersham) supplemented with 5%–10% Lumigen ECL Ultra TMA-6 (Lumigen).

### Fluorescence microscopy, FRAP, and CLEM

8-well µ-Slides (ibidi) coated with 0.4% gelatin were used for all immunofluorescence experiments. ARPE-19, TIME, or TetOn A3 RPE-1 cells were infected with KSHV for 24, 48, or 72 h. Murine10.1 or MEF cells were infected with MHV-68 for 6, 8, or 24 h. For immunofluorescence staining, the cells were washed twice with PBS and fixed with 4% paraformaldehyde (PFA) for 20 min at room temperature (RT). To reduce autofluorescence, free aldehyde groups were neutralized with 50 mM NH_4_Cl for 10 min. Cells were permeabilized with 0.5% Triton X-100 in PBS for 10 min. Samples were blocked in TBS-BG blocking buffer (TBS, 5% glycine, 5% BSA, 0.05% Tween 20, 0.05% sodium azide) for 30 min, incubated with primary antibodies for 1 h and with secondary Alexa Fluor-conjugated antibodies or rhodamine phalloidin (Invitrogen) and Hoechst 33342 (Invitrogen) for 30 min at RT. Fluorescence images were acquired with a Nikon Ti2-based A1 confocal laser scanning microscope (cLSM) using a 1.4 NA 60× Plan Apo objective or a Nikon Ti2-based spinning disk system equipped with a Yokogawa CSU-W1 confocal spinning disk unit and a 1.45 NA 100× Plan Apo objective. Nikon NIS-Elements software was used for image acquisition and ImageJ for image analysis.

For FRAP analysis, U2OS cells were grown on a 35 mm µ-Dish (ibidi) coated with 0.4% gelatin and transfected with ORF61-mCherry or mCherry-Nucleolin expression plasmids. One day later, fluorescence was detected by live-cell imaging using a Nikon A1 cLSM, and FRAP analysis was performed essentially as described (8). Briefly, areas of 1 µm diameter within mCherry-positive structures were half-bleached for 2 s with a 563 nm laser, and the recovery of fluorescence was recorded at two frames per s for 6 min. The relative fluorescence intensity (RFI) over time was calculated as described (58).

For CLEM, ARPE-19 and MEF cells were grown on 35 mm Grid-500 µ-Dishes (ibidi) coated with 0.4% gelatin and infected with KSHV mNeon-ORF61 and MHV-68 ORF61-mNeon, respectively. At 40 hpi (KSHV) or 20 hpi (MHV-68), the cells were fixed with 4% PFA in PBS for 20 min at RT, and nuclei were stained with Hoechst 33342 for 10 min. Fluorescent Z-stacks were acquired with a Nikon A1 cLSM using a 0.75 NA 20× Plan Apo VC objective, deconvolved with Nikon NIS-Elements, and later used for correlation with transmission EM images. Samples were processed for EM as described (59), and at least ten aggregates per sample were analyzed.

KSHV ORF61 colocalization with A3s was quantified using the ImageJ plugin colocalization threshold, and Pearson’s correlation coefficient (r) was calculated for each condition (*n* = 10). The r coefficient gives a score of 1 for perfect colocalization and 0 for no colocalization. For statistical analysis, an unpaired two-tailed Student’s *t*-test was performed.

For quantification of the nuclear A3B fluorescence intensity (%), nuclear and cytoplasmic outlines of the cells (*n* = 12) were defined by thresholding the Hoechst and A3 signal intensities, respectively. The mean nuclear and total A3B intensities were then calculated, corrected for background, and multiplied by the area of the corresponding outlines to obtain the sum fluorescence intensity of the selected area. The sum A3B intensity of the whole cell was defined as 100% for the calculation of the nuclear intensity.

### Viral replication kinetics

For MHV-68 replication kinetics, 3.5 × 10^4^ MEF cells were infected at MOI 0.01 with either MHV-68 ORF61-FLAG or ORF61*mut*IPAM-FLAG in triplicates. The input virus was removed 4 h later, and fresh medium was added. The supernatants were harvested at different times post-infection, stored at −80°C, and titrated on MEF cells. For KSHV replication kinetics, 1.5 × 10^4^ TetOn A3B RPE-1 cells were infected at MOI 0.025 with either KSHV mNeon-ORF61 or mNeon-ORF61*mut*IPAM in triplicates using centrifugal enhancement (1,065 × *g*, 30 min). The input virus was removed 4 h later, and fresh medium containing 1 µM dNTPs was added. To induce A3B overexpression, cells were treated with 1 µg/mL doxycycline. Supernatants were harvested at different times post-infection, stored at −80°C, and titrated on ARPE-19 cells.

### Differential DNA denaturation PCR (3D-PCR)

TetOn A3B RPE-1 cells were infected with KSHV mNeon-ORF61 or mNeon-ORF61*mut*IPAM and either treated with 1 µg/mL doxycycline or left untreated. At 7 dpi, supernatants were collected, and viral DNA was extracted with an innuPREP DNA Mini Kit 2.0 (Innuscreen GmbH) as per the manufacturer’s instructions. The extracted DNA was used as a template for a first-round PCR to amplify a 577 bp fragment of the viral ORF45 with primers 5′-AGGCAATAACTCGTGTGCTTTGTAAAT-3′ and 5′-AAAATCCGTCATCCTGACTAACCCATC-3′ using DreamTaq DNA polymerase (Thermo Fisher Scientific) according to the manufacturer’s protocol. One microliter of the product was then used as a template for a second PCR using primers 5′-AGCACACACGATGAAGAGAGAATGCTT-3′ and 5′-TACCACTGCTACCGGTTTGGGCGTATG-3′ to amplify a 411 bp fragment applying the following PCR conditions: a denaturation temperature gradient from 84.4 to 86.0°C for 5 min followed by 35 cycles of 1 min denaturation at 84.4 to 86.0°C, 30 s annealing at 62°C, 25 s elongation at 72°C, and 10 min final elongation at 72°C. Both PCRs were run on a Biometra TAdvanced Twin 48 thermocycler (Analytik Jena). The PCR products were separated on 1% agarose gels and visualized with ethidium bromide staining.

## Data Availability

All relevant data are contained in the article and its supplemental material.

## SUPPLEMENTAL MATERIAL

Supplemental figures jvi.00789-25-s0001.pdf Figures S1 to S3.

ASM does not own the copyrights to Supplemental Material that may be linked to, or accessed through, an article. The authors have granted ASM a non-exclusive, world-wide license to publish the Supplemental Material files. Please contact the corresponding author directly for reuse.
