# Supplementary material for: Kaposi’s sarcoma-associated herpesvirus ORF61 protein sequesters APOBEC3B in filamentous aggregates
Source: J Virol. 2025 Jun 5;99(7):e00789-25. doi: 10.1128/jvi.00789-25 (PMC12282084; doi:10.1128/jvi.00789-25)
Supplement: Supplemental figures — Figures S1 to S3. [file jvi.00789-25-s0001.pdf]

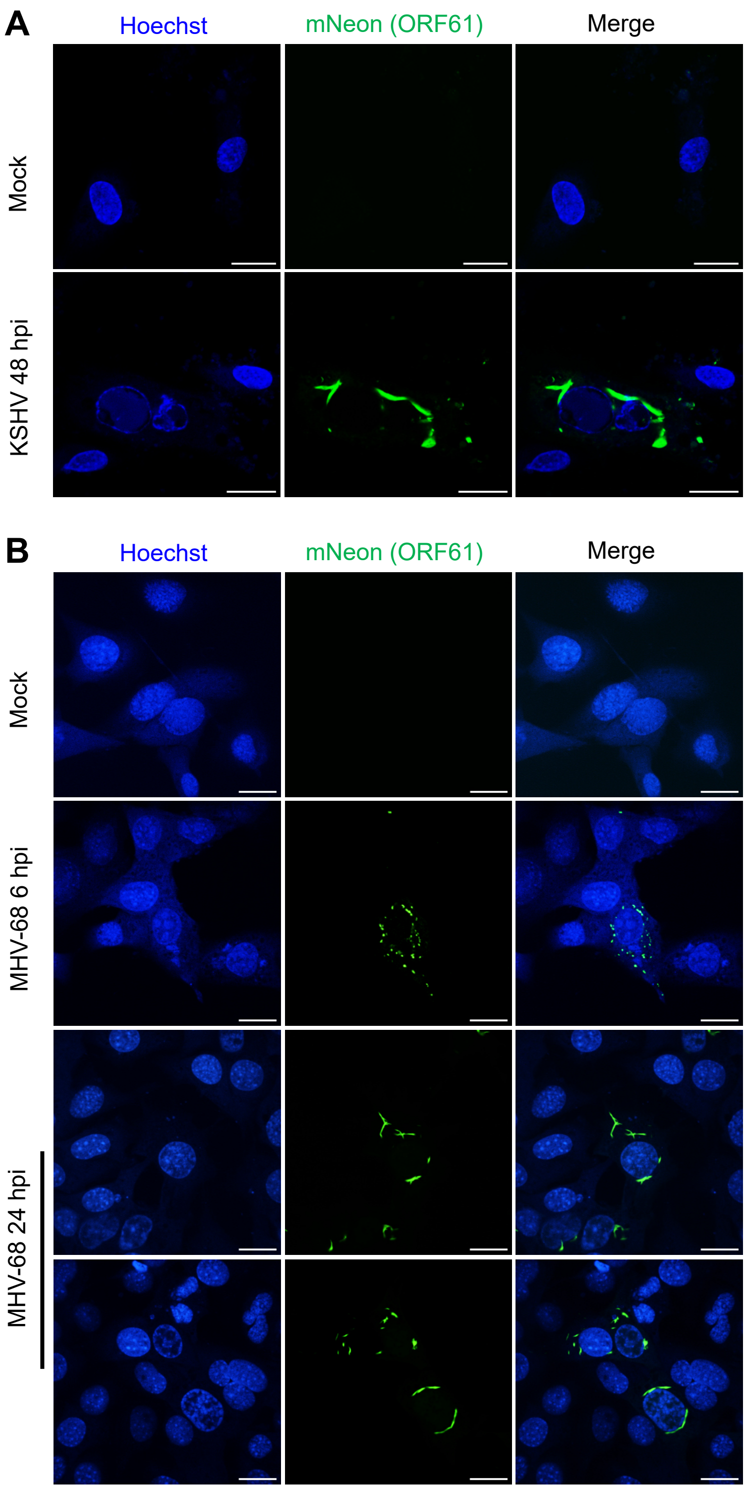


**Figure S1. Formation of rhadinovirus R1 condensates in infected cells is not influenced by the cell type or tag used.** Confocal microscopy images of (A) TIME cells infected with KSHV mNeon-ORF61 and (B) MEF cells infected with MHV-68 ORF61-mNeon. Cells were fixed at the indicated times postinfection and nuclei counterstained with Hoechst 33342. Scale bar, 20 µm.

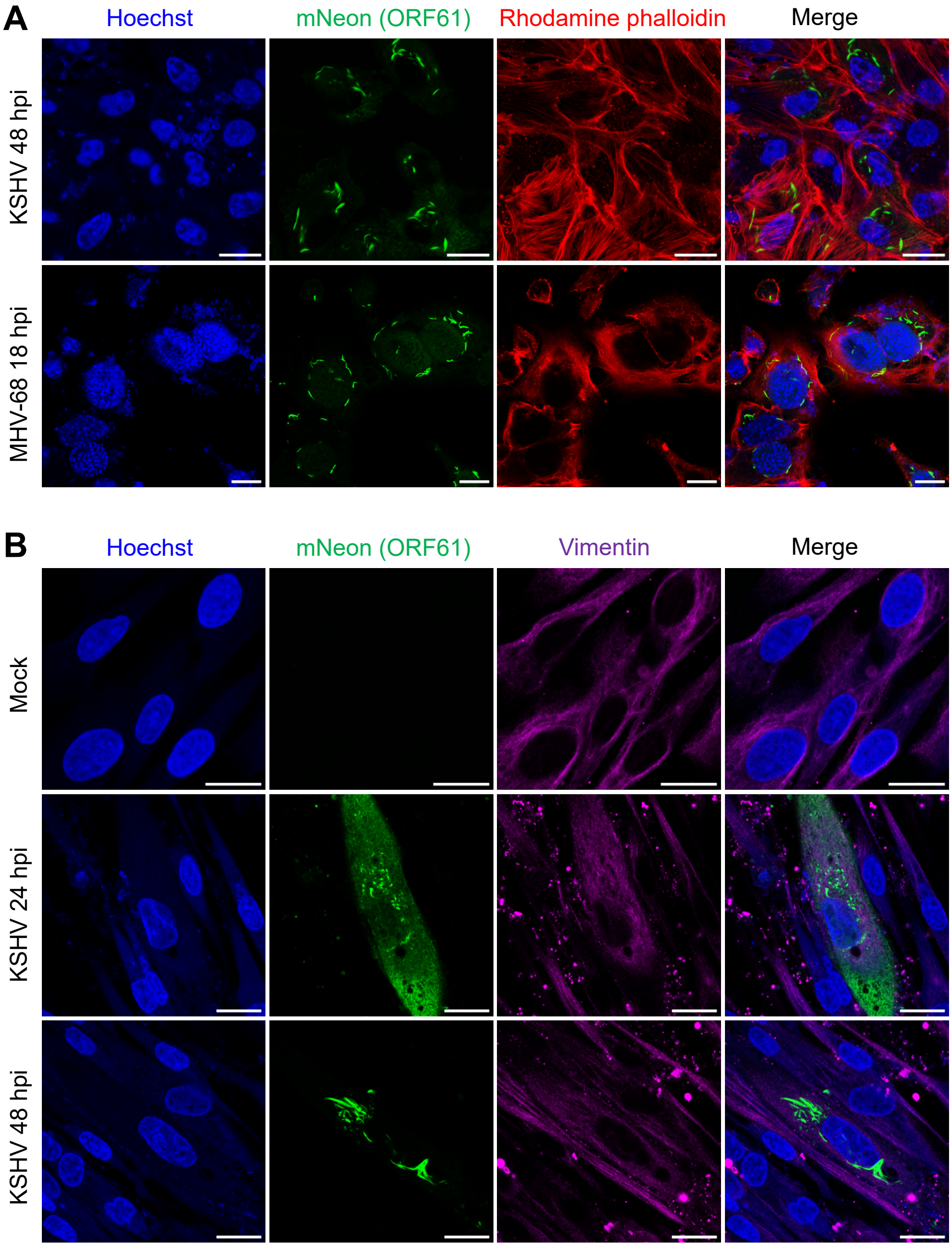


**Figure S2. Rhadinovirus R1 protein aggregates do not contain F-actin or vimentin.** (A) ARPE-19 cells infected with KSHV mNeon-ORF61 and MEF cells infected with MHV-68 ORF61-mNeon were analyzed by cLSM. Cells were fixed at the indicated times postinfection, F-actin was stained with rhodamine phalloidin, and nuclei were stained with Hoechst 33342. (B) HFF cells infected with KSHV mNeon-ORF61, fixed at 24 or 48 hpi, stained with an anti-vimentin antibody and Hoechst 33342, and analyzed by cLSM. Scale bar, 20 µm.

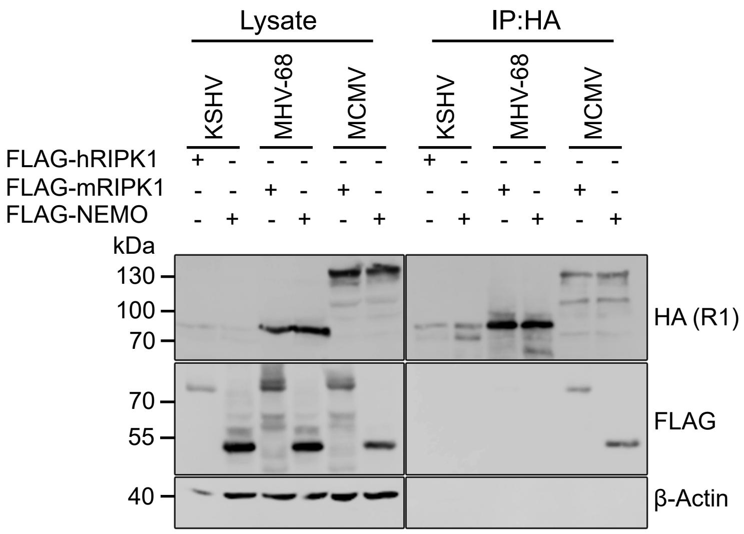


**Figure S3. Unlike the homologous MCMV M45, rhadinovirus R1 proteins do not interact with RIPK1 or NEMO.** HEK-293A cells were transfected with plasmids encoding HA-tagged R1 proteins and FLAG-tagged human or murine RIPK1 or NEMO. At 24 h post-transfection, cells were lysed and R1 proteins precipitated using an anti-HA antibody. Co-precipitating proteins were visualized by immunoblot. MCMV M45 was used as a positive control.
